# NVX-CoV2373-induced T- and B-cellular immunity in immunosuppressed people with multiple sclerosis that failed to respond to mRNA and viral vector SARS-CoV-2 vaccines

**DOI:** 10.3389/fimmu.2023.1081933

**Published:** 2023-07-20

**Authors:** Magdalena Mueller-Enz, Christina Woopen, Georges Katoul Al Rahbani, Rocco Haase, Marie Dunsche, Tjalf Ziemssen, Katja Akgün

**Affiliations:** Center of Clinical Neuroscience, University Hospital Carl Gustav Carus, Dresden University of Technology, Dresden, Germany

**Keywords:** SARS-CoV-2 vaccination, NVX-CoV2373, multiple sclerosis, sphingosine-1phosphate receptor modulators, anti-CD20 therapy, immunomodulation, humoral and T cellular vaccination response

## Abstract

**Importance:**

Immunological response to severe acute respiratory syndrome coronavirus 2 (SARS-CoV-2) vaccination is important, especially in people with multiple sclerosis (pwMS) on immunosuppressive therapies.

**Objective:**

This study aims to determine whether adjuvanted protein-based vaccine NVX-CoV2373 is able to induce an immune response to SARS-CoV-2 in pwMS with inadequate responses to prior triple mRNA/viral vector vaccination.

**Design, setting, and participants:**

We conducted a single-center, prospective longitudinal cohort study at the MS Center in Dresden, Germany. In total, 65 participants were included in the study in accordance with the following eligibility criteria: age > 18 years, immunomodulatory treatment, and insufficient T-cellular and humoral response to prior vaccination with at least two doses of SARS-CoV-2 mRNA (BNT162b2, mRNA-1273) or viral vector vaccines (AZD1222, Ad26.COV2.S).

**Interventions:**

Intramuscular vaccination with two doses of NVX-CoV2373 at baseline and 3 weeks of follow-up.

**Main outcomes and measures:**

The development of SARS-CoV-2-specific antibodies and T-cell responses was evaluated.

**Results:**

For the final analysis, data from 47 patients on stable treatment with sphingosine-1-phosphate receptor (S1PR) modulators and 17 on ocrelizumab were available. The tolerability of the NVX-CoV2373 vaccination was overall good and comparable to the one reported for the general population. After the second NVX-CoV2373 vaccination, 59% of S1PR-modulated patients developed antispike IgG antibodies above the predefined cutoff of 200 binding antibody units (BAU)/ml (mean, 1,204.37 [95% CI, 693.15, 2,092.65] BAU/ml), whereas no clinically significant T-cell response was found. In the subgroup of the patients on ocrelizumab treatment, 23.5% developed antispike IgG > 200 BAU/ml (mean, 116.3 [95% CI, 47.04, 287.51] BAU/ml) and 53% showed positive spike-specific T-cellular responses (IFN-gamma release to antigen 1: mean, 0.2 [95% CI, 0.11, 0.31] IU/ml; antigen 2: mean, 0.24 [95% CI, 0.14, 0.37]) after the second vaccination.

**Conclusions:**

Vaccination with two doses of NVX-CoV2373 was able to elicit a SARS-CoV-2-specific immune response in pwMS lacking adequate immune responses to previous mRNA/viral vector vaccination. For patients receiving S1PR modulators, an increase in anti-SARS-CoV-2 IgG antibodies was detected after NVX-CoV2373 vaccination, whereas in ocrelizumab-treated patients, the increase of antiviral T-cell responses was more pronounced. Our data may impact clinical decision-making by influencing the preference for NVX-CoV2373 vaccination in pwMS receiving treatment with S1PR modulation or anti-CD20 treatment.

## 1 Introduction

The severe acute respiratory syndrome coronavirus 2 (SARS-CoV-2) pandemic has dramatically accelerated the progress in research on viral infections and vaccination. Up until November 2021, four SARS-CoV-2 vaccines had received marketing authorization in the European Union, of which two were based on mRNA and two on viral vector technology. Multiple studies have shown good efficacy for mRNA and viral vector vaccines with regard to the prevention of SARS-CoV-2 symptomatic infection and severe coronavirus disease 2019 (COVID-19) disease courses in the general population (1–7). Immunological analyses provided evidence for humoral and T-cellular responses directed against SARS-CoV-2 (8–18). However, further research revealed decreased or even lacking immunological responses to SARS-CoV-2 vaccination in certain subpopulations, especially in patients who receive immunosuppressive therapies due to autoimmune disease or cancer. Unfortunately, the same patients are at risk for a severe COVID-19 disease course due to their immunotherapy. Immunosuppressive treatments are used in people with multiple sclerosis (pwMS) for disease modification. Two categories of MS drugs have been shown to impair immune responses to mRNA and viral vector vaccination. First, sphingosine-1-phosphate receptor (S1PR) modulators, which prevent lymphocyte egress from the lymph nodes, have been shown to impair humoral and T-cellular responses to SARS-CoV-2 vaccination (19–21). Second, treatment with monoclonal anti-CD20 antibodies limited patients’ ability to mount adequate humoral responses to SARS-CoV-2 vaccines (22–27). In December 2021, the protein-based adjuvanted SARS-CoV-2 vaccine NVX-CoV2373 received conditional marketing authorization in the European Union. We aimed to clarify whether NVX-CoV2373 can induce SARS-CoV-2-specific T- and B-cell immunity in pwMS on S1PR modulator and anti-CD20 treatment who failed to respond to initial triple vaccination with mRNA or vector vaccines.

## 2 Materials and methods

### 2.1 Patients and informed consent

**Figure 1:**
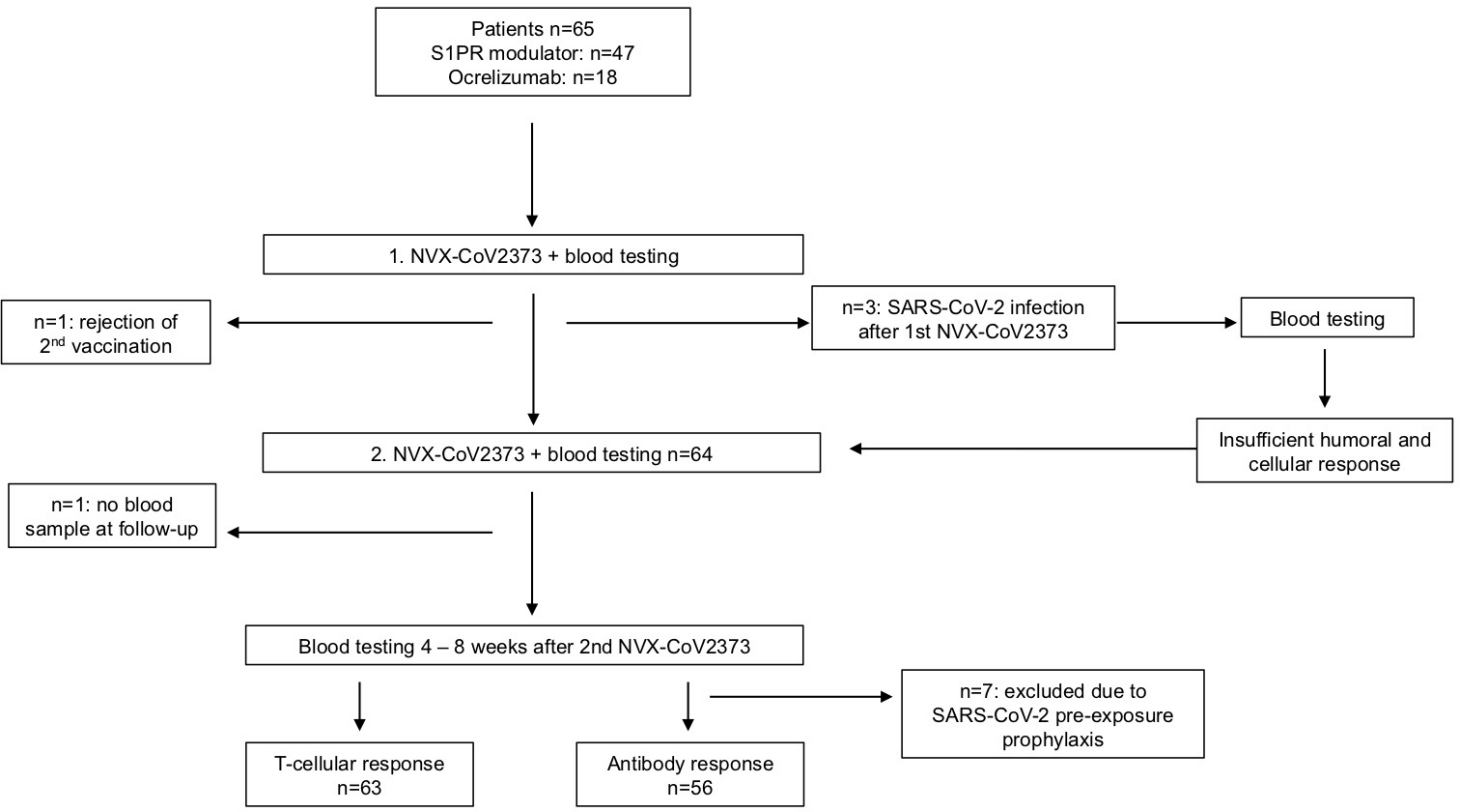
Enrollment and outcomes. The full analysis set included all participants who presented an insufficient T- and B-cell response to prior vaccination with at least two doses of SARS-CoV-2 mRNA or viral vector vaccines. One patient each was excluded after the first and second vaccinations with NVX-CoV2373. Seven patients were excluded from antibody testing because of pre-exposure prophylactic treatment with tixagevimab/cilgavimab.

We conducted a prospective longitudinal cohort study among pwMS at the MS Center in Dresden, Germany (**Figure 1**). Data of 975 MS patients on different immunomodulatory therapies vaccinated against SARS-CoV-2 between April 2021 and April 2022 were screened for eligibility. In total, 167 of those patients met the predefined criteria: age > 18 years, immunomodulatory treatment, and insufficient cellular and humoral response to prior vaccination with at least two doses of mRNA (BNT162b2, mRNA-1273) or viral vector vaccines (AZD1222, Ad26.COV2.S) against SARS-CoV-2. Of these patients, 65 consented to be vaccinated with NVX-CoV2373 and were included in the study. Standardized testing for SARS-CoV-2-specific T- and B-cellular responses was done using manufacturer-certified analysis. Insufficient immune response was defined as a negative T-cellular response (interferon (IFN)-gamma release to SARS-CoV-2 antigen 1 (Ag1) and antigen 2 (Ag2) <0.15 IU/ml) and anti-SARS-CoV-2 spike protein IgG antibodies < 200 binding antibody units (BAU)/ml. The cutoff level for antibody titer was defined using the lower range of a cohort of *n* = 62 MS patients without disease-modifying therapy that mounted a B-cellular response of mean 968.51 (199.8; > 2,080 (min–max)) BAU/ml, 125.14 ± 69.31 (mean ± SD) after primary vaccination with mRNA vaccines.

Results from previous testing on T- and B-cellular responses were available after initial two doses of mRNA/viral vector vaccination (primary vaccination, T-2) and after booster vaccination with mRNA vaccine (T-1). Patients were vaccinated with NVX-CoV2373 intramuscularly twice, at baseline and 3 weeks later. Blood samples were collected on a date before the first NVX-CoV2373 (T0), 3 weeks after the first and at the date of the second NVX-CoV2373 vaccination (T1), and 4 to 8 weeks after the second NVX-CoV2373 vaccination (T2, **Figure 1**). The time between vaccinations and blood collection is defined in **Table 1**. Patients were asked to complete a standardized questionnaire to document adverse events after each NVX-CoV2373 vaccination. The study was approved by the institutional review board of the University Hospital Dresden. Patients gave their written informed consent.

**Table 1:** Time between vaccination and measurement of SARS-CoV-2-specific immune response.

	S1PR modulation	Ocrelizumab
T-2	96.58 ± 53.25	109.92 ± 77.75
T-1	63.14 ± 42.12	65.72 ± 44.44
T0	148.59 ± 43.31	152.50 ± 34.49
T1	22.68 ± 4.87	23.12 ± 5.40
T2	30.37 ± 5.26	31.94 ± 5.45

Days in mean ± SD between vaccination and blood testing. T-2, after two doses of mRNA/viral vector vaccination (primary vaccination); T-1, after booster vaccination with mRNA vaccine; T0, follow-up after booster vaccination with mRNA vaccine and at the date of the first NVX-CoV2373 vaccination; T1, 3 weeks after the first and at the date of the second NVX-CoV2373 vaccination; T2, 4 to 8 weeks after the second NVX-CoV2373 vaccination. Differences between groups were not statistically significant.

### 2.2 Analysis of SARS-CoV-2-specific T-cell response

Lithium-heparin blood samples were taken and freshly prepared after collection. The certified SARS-CoV-2 QuantiFERON test (Qiagen, Hilden, Germany) was used to measure the CD4 and CD8 IFN-gamma secretion after stimulation with SARS-CoV-2 spike protein peptide pools (27). The tube with Ag1 contained CD4+ epitopes from the S1 subunit 1 whereas the tube with Ag2 contained CD4+ and CD8+ epitopes from the S1 and S2 subunit of the spike protein. Blood samples were incubated for 16 to 24 h with Ag1, Ag2, or with mitogen as a positive control. IFN-gamma release in the negative control of each sample was subtracted from responses to Ag1/2. The positivity cutoff was predefined as 0.15 IU/ml based on the manufacturer’s instructions.

### 2.3 Detection of SARS-CoV-2-specific antibodies

The collected serum samples were directly prepared for measurement. The LIAISON^®^ SARS-CoV-2 TrimericS IgG assay (DiaSorin, Saluggia, Italy) was used in a certified laboratory to quantify SARS-CoV-2-specific antibodies. The seropositivity cutoff was predefined as 33.8 BAU/ml based on the manufacturer’s instructions. The lower detection limit was 4.81 BAU/ml, values < 4.81 BAU/ml were set to half the detection limit, i.e., 2.405 BAU/ml. The upper detection limit was 2,080 BAU/ml, patients with values above this threshold were set to 2,081 BAU/ml.

### 2.4 Complete blood count and immune cell phenotyping

Standardized blood testing was performed for complete blood cell counts and peripheral cell subsets at the Institute of Clinical Chemistry and Laboratory Medicine, University Hospital Dresden, Germany. Immune cell phenotyping was done by flow cytometry using fluorescence-labeled anti-CD3, anti-CD4, anti-CD8, and anti-CD19 (BD Biosciences, Heidelberg, Germany) according to the manufacturer’s instructions and evaluated on FACSCanto II. Negative controls included directly labeled or unlabeled isotype-matched irrelevant antibodies (BD Biosciences).

### 2.5 Statistical analysis

Data were analyzed descriptively, calculating means and standard deviations (SD) for the total study sample and relevant subgroups. For the classification of the distribution of outcomes, Q–Q plots were created. IFN-gamma release and antibody titers were analyzed *via* generalized linear models (GLM) with gamma-log link functions and reported as model estimates (mean and 95% confidence interval (CI)). Time (repeated samplings), age (years), sex, disease duration (days), treatment (S1PR vs. anti-CD20), treatment duration (days), disability (via Expanded Disability Status Scale), previous SARS-CoV-2 infection, the time between prior vaccination and sampling, prophylactic treatment with tixagevimab/cilgavimab, and interactions between time and treatment, time and previous SARS-CoV-2 infection, time and prophylactic treatment with tixagevimab/cilgavimab, time and disability, as well as time, treatment, and disability served as fixed factors. The Sidak correction for pairwise testing was applied. Spearman’s rho was used to calculate correlations.

## 3 Results

### 3.1 Study population and patient characteristics

We included 65 pwMS with an age of 49.7 ± 11.37 years (mean ± SD). The study population presented a disease duration of 14.1 ± 7.36 years (mean ± SD) with a relapsing disease course in most of the patients (**Table 2**). A total of 47 (72.3%) patients were on stable treatment with S1PR modulators, fingolimod or siponimod, and 18 (27.7%) patients were on stable treatment with ocrelizumab.

**Table 2:** Patient characteristics.

Age (years (mean, SD))	49.70 (11.37)
Female (no, %)	35 (53.85%)
Disease duration (years (mean, SD))	14.1 (7.36)
Disease course (no, %)	
RRMS	55 (84.62%)
PPMS	2 (3.08%)
SPMS	8 (12.31%)
Vaccination type (no, %)	
2× mRNA/VVV	2 (3.08%)
3× mRNA/VVV	61 (93.84%)
Infection + 2× mRNA/VVV	2 (3.08%)
Treatment (no, %)	
S1PR modulator^a^	47 (72.31%)
Ocrelizumab	18 (27.69%)
Treatment duration (days (mean, min–max))	
S1PR modulator	2,223 (204–3,959)
Ocrelizumab	901 (154–1,767)

a Fingolimod n = 43; siponimod n = 4; RRMS, relapsing–remitting multiple sclerosis; PPMS, primary progressive multiple sclerosis; SPMS, secondary progressive multiple sclerosis; VVV, viral vector vaccine; S1PR, sphingosine-1-phosphate receptor.

Most of the patients (94%) were triple vaccinated; fewer were vaccinated twice (3%) or had a combination of two vaccine doses and status post-SARS-CoV-2 infection (3%) prior to the vaccination with NVX-CoV2373 (**Table 2**). The primary immunization was completed with mRNA vaccines in most patients (76.9%), while a smaller group (10.8%) was initially vaccinated with viral vector vaccines. Primary immunization with one dose each of mRNA and viral vector vaccine was performed in six patients (9.2%), and with one dose of mRNA vaccine in combination with a SARS-CoV-2 infection in two patients (3.1%). All patients with a third vaccination received mRNA vaccines (BNT162b2, mRNA-1273).

Previous SARS-CoV-2 infection was reported for 17 patients (ocrelizumab: *n* = 6, S1PR modulators: *n* = 11). Three patients reported infection with SARS-CoV-2 between the first and second doses of NVX-CoV2373. An additional blood sample 2–4 weeks after the infection was taken to assess the immune response (**Figure 1**). Since all three patients still lacked a sufficient immune response to SARS-CoV-2, the second dose of NVX-CoV2373 was administered.

### 3.2 Tolerability of NVX-CoV2373 vaccination

Patients were asked to complete a standardized questionnaire to document side effects (**Table 3**). The questionnaire was completed by 59 patients after the first and by 57 patients after the second dose. Overall, approximately half of the patients showed side effects after NVX-CoV2373 vaccination (54% after the first and 58% after the second dose), the most frequent being pain at the injection site, headaches, and fatigue. Four patients complained about the aggravation of MS symptoms after the first dose and three patients after the second dose. The symptoms included neuralgia, paresthesia, and impaired vision, coordination, or mobility. One patient refused the second dose due to symptoms including pain at the injection site, headache, melalgia, fever, shivering, fatigue, as well as impaired mobility, coordination, and physical condition for more than 3 weeks. Two patients needed to be hospitalized shortly after the second vaccination due to cardiac and urological diseases. A certain correlation between the illnesses and the vaccination could not be found.

**Table 3:** Vaccination tolerability.

Side effects after NVX-CoV2373	First dose(*n*, %)	Second dose(*n*, %)
Overall	32 (54.24%)	33 (57.89%)
Pain at the injection site	20 (33.90%)	18 (31.58%)
Headache	15 (25.42%)	14 (24.56%)
Melalgia	7 (11.86%)	9 (15.79%)
Fever	3 (5.08%)	0 (0%)
Shivering	2 (3.39%)	2 (3.51%)
Fatigue	17 (28.81%)	18 (31.58%)
Aggravation of MS symptoms	4 (6.78%)	3 (5.26%)
Others	3 (5.08%)	6 (10.53%)

Patient number first dose n = 59; second dose n = 57.

### 3.3 NVX-CoV2373 vaccination during S1PR modulation

**Figure 2:**
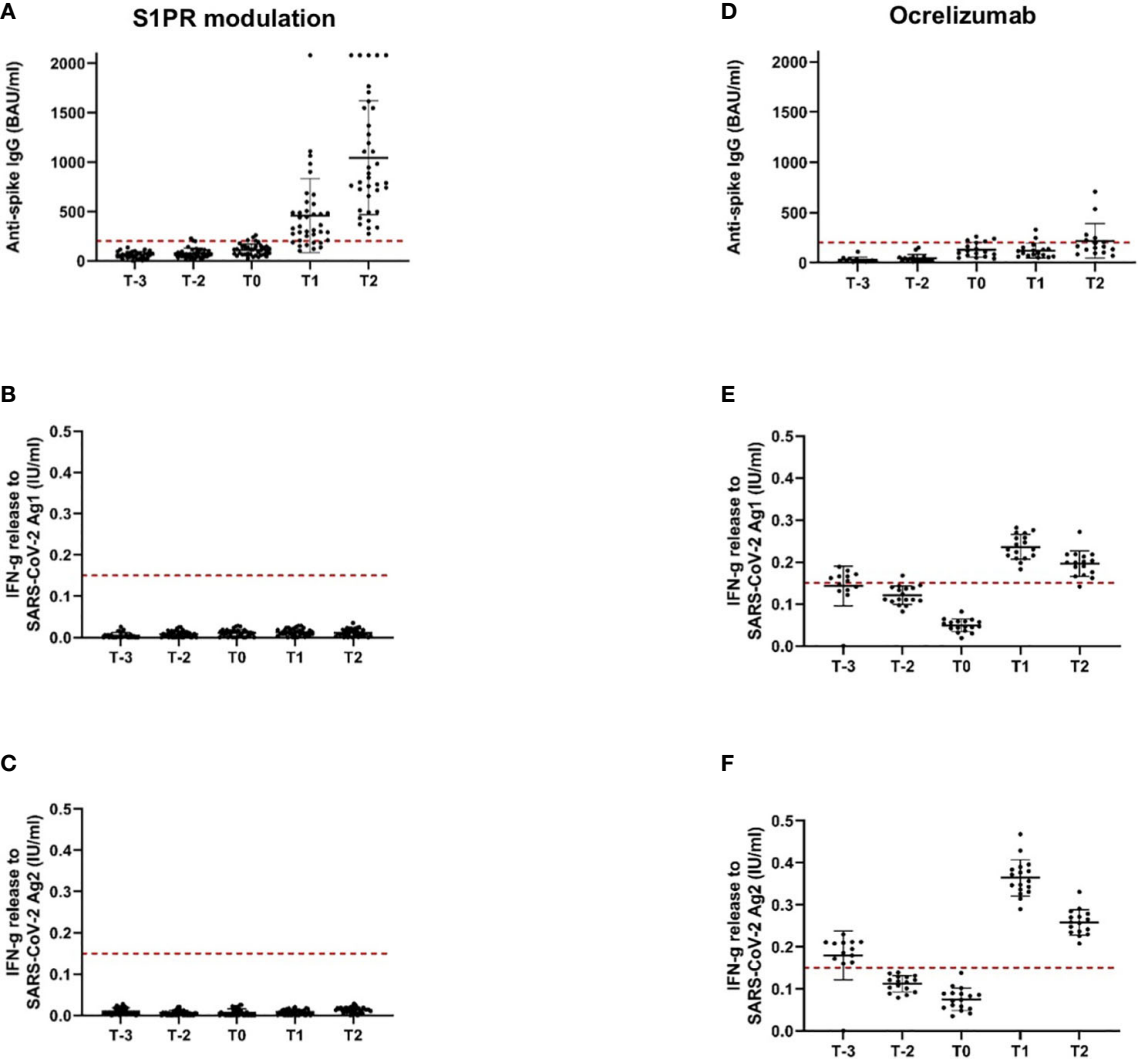
Antispike IgG antibody and T-cellular response to SARS-CoV-2 vaccination in pwMS under S1PR modulation and ocrelizumab. Immune responses to primary and booster mRNA/viral vector vaccination, as well as to vaccination with NVX-CoV2373 in pwMS on S1PR modulation **(A–C)** and ocrelizumab therapy **(D–F)** are depicted. Scatter plots with means and standard deviation are presented and were calculated *via* the generalized linear model, considering the maximum detection limit of the performed assays. Five time points are defined: T-2, after primary immunization with mRNA/viral vector vaccines; T-1, after booster vaccination with mRNA vaccines; T0, baseline measurement on the day of the first NVX-CoV2373 vaccination; T1, 3 weeks after the first NVX-CoV2373 vaccination on the day of the second vaccination; T2, follow-up 4 to 8 weeks after the second vaccination with NVX-CoV2373. Cutoff lines according to the predefined criteria for insufficient immune response (antispike IgG antibodies < 200 BAU/ml and interferon-gamma release to Ag1 and Ag2 < 0.15 IU/ml) are delineated (red, dotted lines).

Our study included 47 patients on treatment with S1PR modulators (fingolimod *n* = 43, siponimod *n* = 4; **Table 1**). Patients were on stable therapy with a mean treatment duration of 2,223 days at the time point of NVX-CoV2373 vaccination. Based on the inclusion criteria, SARS-CoV-2-specific antibodies and T-cell response were below positivity cutoff after primary and booster mRNA/viral vector vaccination and before the first NVX-CoV2373 dose independent of mRNA versus viral vector vaccine use (T-2, T-1, T0, **Figures 2A-C**). The time between the first dose of vaccination and booster vaccination (T-1) was 215 ± 31 days and between booster (T-1) and the first dose of NVX-CoV2373 was 149 ± 43 days. Three weeks after the first NVX-CoV2373, no patient presented a positive T-cellular response, whereas 32% of patients responded with positive antispike IgG antibodies (536.48 [286.99, 1,002.83] BAU/ml; mean, 95% CI; T1, **Figures 2A-C**; **Table 4A**, higher than predefined cutoff (> 200 BAU/ml). After the second vaccination dose, 59% of patients responded with increased antispike IgG antibody titers (1,204.37 [693.15, 2,092.65] BAU/ml; mean, 95% CI; T2, **Figure 2A**; **Table 4A**. The results of seven patients were not taken into consideration due to pre-exposure prophylactic antibody treatment that was started between T1 and T2. Regarding the T-cellular response, only two (4.3%) patients presented elevated levels after the second dose of NVX-CoV2373 (IFN-gamma release to Ag1 and Ag2 0.002 [0, 0.015] IU/ml; mean, 95% CI; **Figures 2B, C**; **Table 4A**. There was a moderate correlation between IFN-gamma release from Ag1 and the overall level of lymphocytes (rho = 0.332). No further significant correlations between the titer of antibody response and lymphocyte levels (including CD3, CD4, CD8, and CD19 lymphocyte subtypes) were found. Previous infection with SARS-CoV-2 led to higher antispike IgG antibody levels, whereas no significant correlation between previous SARS-CoV-2 infection and T-cellular response could be noted. Differences in absolute lymphocyte count and its subsets as the underlying reason for the variation in the SARS-CoV-2-specific response could be excluded: patients were on stable treatment during the whole observation period and presented a similar range of lymphocyte, CD3, CD4, CD8, and CD19 cell counts at the time point of primary and booster vaccination with mRNA and viral vector vaccines as well as NVX-CoV2373 vaccination (**Table 5**).

**Table 4:** Model estimates of T- and B-cell response generated *via* GLM.

	T-2	T-1	T0	T1	T2
A					
S1P					
Antispike IgG (BAU/ml)	24.2 (45.9; 12.7)	111.2 (170.9; 72.3)	108.8 (245.8; 48.2)	536.5 (1,002.8; 289.9)	1,204.4 (2,092.6; 693.1)
IFN-g release to Ag1 (IU/ml)	0.007 (0.03; 0.0)	0.002 (0.02; 0.0)	0.0 (0.01; 0.0)	0.001 (0.01; 0.0)	0.002 (0.02; 0.0)
IFN-g release Ag2 (IU/ml)	0.018 (0.04; 0.0)	0.004 (0.01; 0.0)	0.0 (0.02; 0.0)	0.001 (0.01; 0.0)	0.002 (0.02; 0.0)
Ocrelizumab					
Antispike IgG (BAU/ml)	6.3 (25.1; 1.6)	30.0 (50.2; 18.0)	75.1 (296.4; 19.0)	76.3 (204.6; 28.5)	116.3 (287.5; 47.0)
IFN-g release to Ag1 (IU/ml)	0.117 (0.37; 0.06)	0.135 (0.2.1; 0.08)	0.052 (0.09; 0.02)	0.234 (0.31; 0.17)	0.196 (0.31; 0.11)
IFN-g release to Ag2 (IU/ml)	0.215 (0.36; 0.11)	0.125 (0.16; 0.09)	0.07 (0.12; 0.03)	0.345 (0.46; 0.26)	0.235 (0.37; 0.14)
B					
Ocrelizumab before 30–89 days					
Antispike IgG (BAU/ml)			43.0 (72.8; 25.4)	37.9 (65.3; 22.1)	36.6 (73.3; 18.3)
IFN-g release to Ag1 (IU/ml)			0.074 (0.12; 0.04)	0.215 (0.3; 0.15)	0.139 (0.23; 0.08)
IFN-g release to Ag2 (IU/ml)			0.133 (0.23; 0.07)	0.323 (0.45; 0.23)	0.161 (0.25; 0.09
Ocrelizumab last before > 90 days					
Antispike IgG (BAU/ml)			109.5 (274.0; 43.7)	142.1 (264.7; 76.3)	213.4 (457.9; 99.5)
IFN-g release to Ag1 (IU/ml)			0.012 (0.02; 0.001)	0.224 (0,37; 0.15)	0.185 (0.29; 0.11)
IFN-g release to Ag2 (IU/ml)			0.04 (0.09; 0.005)	0.42 (0.59; 0.29)	0.308 (0.49; 0.18)

Mean and 95% confidence interval (CI) estimated from the generalized linear models (GLM).

**Table 5:** Lymphocyte counts at time points of vaccination.

Treatment	Time point	Lymphocyte count (GPT/ml) (mean, 95% CI)	CD3+ count (GPT/ml) (mean, 95% CI)	CD4+ count [GPT/ml] (mean, 95% CI)	CD8+ count (GPT/ml) (mean, 95% CI)	CD19+ count (GPT/ml] (mean, 95% CI)
S1P (*n* = 47)	First/second mRNA/VVV	0.455 (0.305 – 0.679)	0.216 (0.125–0.375)	0.037 (0.022–0.063)	0.133 (0.080–0.220)	0.010 (0.008–0.012)
	Third mRNA/VVV	0.421 (0.356–0.498)	0.212 (0.158–0.283)	0.049 (0.029–0.082)	0.137 (0.101–0.186)	0.016 (0.011–0.023)
	First/second NVX-CoV2373	0.449 (0.376–0.535)	0.227 (0.174–0.297)	0.062 (0.036–0.109)	0.141 (0.110–0.180)	0.020 (0.011–0.036)
OCR(*n* = 18)	First/second mRNA/VVV	1.051 (0.662–1.667)	0.695 (0.378–1.278)	0.284 (0.154–0.524)	0.190 (0.108–0.333)	0.001 (0.001–0.001)
	Third mRNA/VVV	1.068 (0.878–1.297)	0.812 (0.627–1.052)	0.584 (0.427–0.798)	0.233 (0.167–0.325)	0.003 (0.001-0.007)
	First/second NVX-CoV2373	1.099 (0.880–1.372)	0.883 (0.679–1.147)	0.730 (0.495–1.075)	0.260 (0.194–0.347)	0.003 (0.001–0.008)

S1P, sphingosine-1-phosphate receptor modulation; OCR, ocrelizumab; VVV, viral vector vaccine.

### 3.4 NVX-CoV2373 vaccination during ocrelizumab

**Figure 3:**
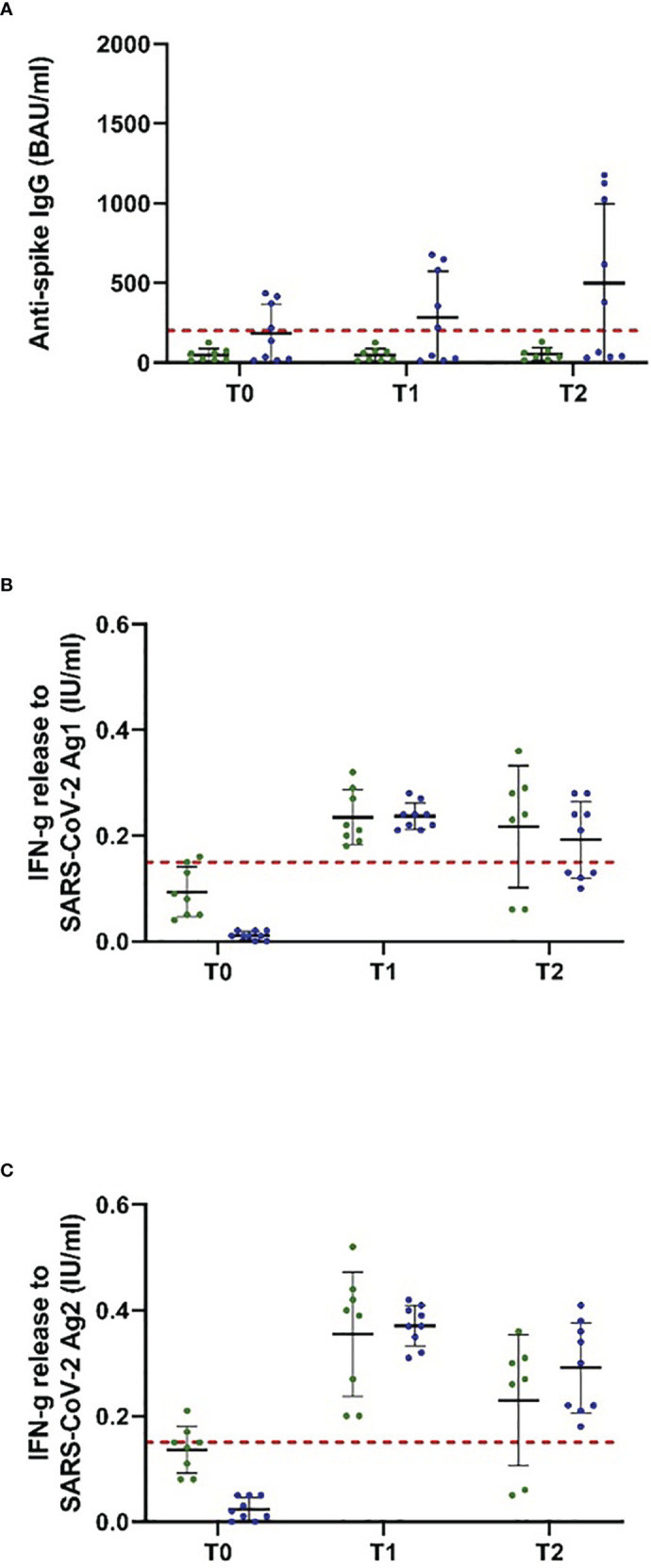
Antispike IgG antibody and T-cellular response to NVX-CoV2373 vaccination in people with MS, depending on the last ocrelizumab infusion. Patients on ocrelizumab were categorized into two subgroups according to the time between the first vaccination with NVX-CoV2373 and the last treatment cycle (30–89 days, green; > 90 days, blue). Scatter plots with means and standard deviation are presented and were calculated *via* the generalized linear model, considering the maximum detection limit of the performed assays. **(A)** B cellular response defined by antispike IgG antibodies and **(B, C)** T cellular response defined by IFN-g (interferon-gamma) release to Ag1 and Ag2 are presented. Three time points are defined: T0, baseline measurement on the day of the first NVX-CoV2373 vaccination; T1, 3 weeks after the first vaccination on the day of the second vaccination; T2, follow-up 4 to 8 weeks after the second vaccination with NVX-CoV2373. Cutoff lines according to the predefined criteria for insufficient immune response (antispike IgG antibodies < 200 BAU/ml and interferon-gamma (IFN-g) release to Ag1 and Ag2 < 0.15 IU/ml) are delineated (red, dotted lines).

The study population comprised 18 patients on ocrelizumab therapy. Infusions were administered at a dose of 600 mg every 6 months. Patients were on ocrelizumab treatment on average for 901 days (**Table 2**). No included patient exceeded the predefined level of antispike IgG antibodies (> 200 BAU/ml) after primary or booster vaccination according to the inclusion criteria. Following booster immunization with mRNA vaccines, 44.4% of patients showed a positive T-cellular response to Ag 1/2 (IFN-gamma release to Ag 1 0.14 [0.08, 0.21]; Ag2 0.13 [0.09, 0.16] IU/ml, mean, 95% CI); T-1, **Figures 2D-F**; **Table 4B** that decreased below positivity cutoff during follow-up and before vaccination with NVX-CoV2373. Again, the effect on antibody or T-cellular response of primary and initial booster vaccination was independent of mRNA versus viral vector vaccine use. The time between the first dose of vaccination and booster vaccination (T-1) was 219 ± 34 days, and between booster (T-1) and the first dose of NVX-CoV2373, it was 153 ± 35 days. Vaccination was performed earliest, at 4 weeks after the last ocrelizumab infusion, except for one patient who received the first NVX-CoV2373 dose 15 days after the last infusion. The average duration between the last infusion and the first vaccination was 96.6 days (15 min–299 max). One patient refused the second dose of NVX-CoV2373 due to side effects so data from 17 patients were available for the final analysis. On the day of the first NVX-CoV2373 vaccination, SARS-CoV-2-specific antibody and T-cellular responses were below the positivity cutoff (T0, **Figures 2D-F**; **Table 4B**. Even though patients on ocrelizumab demonstrated lower antibody levels than patients on S1PR modulatory therapy, antibody titers also increased after vaccination with NVX-CoV2373. SARS-CoV-2-specific antibody levels higher than the predefined cutoff of 200 BAU/ml were reached by 11% of patients after the first and 23.5% of patients after the second dose (T1, 76.32 [28.47, 204.57]; T2, 116.3 [47.04, 287.51] BAU/ml; mean, 95% CI; **Figure 2D**; **Table 4B**. The increase was highest in patients who had received the last ocrelizumab infusion more than 90 days before vaccination (**Figure 3A**; **Table 4B**. Of ocrelizumab-treated patients, 77% responded with SARS-CoV-2-specific IFN-gamma release by CD4 or CD8 T cells after the first and 53% after the second NVX-CoV2373 (T1, IFN-gamma release to Ag1 0.23 [0.17, 0.31]; Ag2 0.35 [0.26, 0.46] IU/ml; T2, IFN-gamma release to Ag1 0.2 [0.11, 0.31]; Ag2 0.24 [0.14, 0.37]; mean, 95% CI, **Figures 2E, F**; **Table 4B**. However, the T-cellular response was not different in patients who were vaccinated more or less than 90 days after the last ocrelizumab infusion (**Figures 3B, C**; **Table 4B**. No significant correlations between antiviral humoral/T-cellular response and lymphocyte subtypes, especially CD19 lymphocyte count, were found. Also, in ocrelizumab-treated patients, previous SARS-CoV-2 infection was associated with higher antispike IgG antibody levels, but no impact on SARS-CoV-2-specific T-cell response. Lymphocyte counts and especially CD19 subset counts were comparable at time points of primary and booster vaccination with mRNA/viral vector vaccines as well as NVX-CoV2373 vaccination (**Table 5**).

## 4 Discussion

In this study, we present that vaccination with NVX-CoV2373 was able to elicit an immune response to SARS-CoV-2 in pwMS receiving treatment with S1PR modulators or ocrelizumab who had previously mounted insufficient responses to mRNA and/or viral vector vaccination. The tolerability of NVX-CoV2373 vaccination in our cohort and general side effects were mostly similar to those reported in the general population (28).

Our observation raises the question of why immunization with the subunit vaccine NVX-CoV2373 was able to promote immune responses in cases where mRNA/viral vector vaccines had failed. A possible explanation would be that the vaccines contain or encode different antigens, leading to a different degree of immunogenicity during S1PR modulation and ocrelizumab treatment. Both mRNA vaccines (BNT162b2, mRNA-1273) encode the full-length SARS-CoV-2 spike protein. Viral vector vaccines (AZD1222, Ad26.COV2.S) consist of adenovirus vectors that carry the genetic information for the spike protein. In all cases, the vaccinee’s cells transcribe and/or translate the mRNA or DNA and present fragments of the produced spike protein on their surface in order to stimulate an immune response. The subunit vaccine NVX-CoV2373 consists of a recombinant complete spike protein containing genetic modifications meant to stabilize the protein in its prefusion conformation and to impede proteolytic degradation (29). In this case, the recombinant spike protein itself is internalized by the vaccinee’s cells and degraded intracellularly, before peptides are presented on the surface. Since all vaccines therewith either encode or consist of the complete spike protein, the presented SARS-CoV-2 antigens should be mostly equivalent among the different vaccines. However, it is conceivable that the conformation of the spike protein is different if endogenous cells have to transcribe and/or translate the genetic information encoded by the vaccine first. This might lead to slightly different epitopes presented on the cell surface, with NVX-CoV2373 antigens possibly being more immunogenic in pwMS during S1PR or anti-CD20 treatment than antigens presented after mRNA or viral vector vaccination.

A different hypothesis is that a nonantigen component of NVX-CoV2373 renders the vaccine more immunogenic in S1PR-modulated and ocrelizumab-treated pwMS. The responsible component might be the adjuvant as NVX-CoV2373 contains Matrix M whereas the other vaccines are unadjuvanted. Referring to vaccination studies before the SARS-CoV-2 pandemic in S1PR-modulated participants does not paint a completely clear picture of the role of adjuvants in vaccination success. Concerning vaccination with aluminum hydroxide-adjuvanted tetanus toxoid (TT) vaccine and neoantigen keyhole limpet hemocyanin (KLH), two studies showed similar humoral response rates to these vaccines during S1PR modulation compared to untreated participants, however partly encompassing reduced antibody titers (30, 31). As for unadjuvanted pneumococcal polysaccharide (PPV-23) vaccination, one study demonstrated normal humoral vaccination responses under siponimod, whereas another study showed an adequate response rate, but again reduced antibody levels under fingolimod (30, 32). Most inactivated influenza vaccines are unadjuvanted, but some do contain adjuvants. The one study explicitly stating that an unadjuvanted influenza vaccine a was used presented a reduced response rate to vaccination under fingolimod (33). The other studies on influenza vaccination partly showed unimpaired and partly diminished humoral response rates during S1PR modulation (31, 32, 34, 35). In summary, studies analyzing responses to adjuvanted as well as unadjuvanted vaccines other than SARS-CoV-2 reached divergent conclusions, indicating partly maintained and partly decreased vaccination responses during S1PR modulation for both vaccine types.

The lack of comparators to our investigated cohorts is of relevant importance. Besides vaccine-specific immunological characteristics between mRNA, viral vector, and protein-based vaccines booster effects by additional vaccinations may also impact the degree of the SARS-CoV-2-specific immune response. During the COVID-19 pandemic, different recommendations were made regarding SARS-CoV-2 vaccinations, especially in immune-modulated and immunosuppressed patients. Booster vaccinations using mRNA vaccines are effective in a proportion of patients that were seronegative after primary vaccination (36). Up to now, no data are available beyond three mRNA or viral vector vaccinations in S1PR modulator- or ocrelizumab-treated patients, but different reports ask for strategies to advise patients with negative immune responses after three doses (37). In Germany, a A fourth vaccination is recommended in selected patient groups (old and immunosuppressed) since 10/2022. Since approval of NVX-CoV2373 in Europe selected data are available that prove efficacy and immunogenicity in healthy people. Although the presented studies are only partially comparable to our cohort, data on humoral immune responses that are reached after primary vaccination in healthy people are comparable or even higher in contrast to our S1PR-modulated patients (38–40). Only small studies compare mRNA vaccines and protein-based vaccines after primary vaccination, suggesting no or only mild superiority in the induction of T-cellular and humoral response including SARS-CoV-2-neutralizing antibodies of mRNA vaccines (41–43). Current studies suggest heterologous prime-booster strategies to increase SARS-CoV-2-specific immunogenicity and effectiveness and support the idea of change in vaccination type in case of negative immune response after primary or even booster vaccination, especially in patient groups at risk (44). However, especially booster vaccination with NVX-CoV2373 after primary vaccination with mRNA vaccines could not increase immunogenicity compared to other vaccine platforms in healthy people (45).

Regarding prepandemic research on vaccination responses during ocrelizumab treatment, the VELOCE study showed decreased humoral responses to vaccination with adjuvanted (TT, KLH) as well as nonadjuvanted vaccines (PPV-23). Nevertheless, ocrelizumab-treated patients did meet the criteria for seroprotection or mounted a considerable increase in antibody titers after vaccination, indicating an at least partly remaining capacity to respond to vaccination. T-cellular vaccination responses were not analyzed in this trial (46). NVX-CoV2373 is the first licensed vaccine using Matrix M as an adjuvant. Matrix M consists of saponin extracted from the *Quillaja saponaria* Molina tree; its mechanism of action is unknown. However, it has been shown that it enhances the production of vaccine-neutralizing antibodies and leads to a CD4 T helper type 1-skewed response (47–49). Possibly, Matrix M is also responsible for the vaccination response observed in pwMS previously lacking adequate responses to SARS-CoV-2 mRNA and viral vector vaccination. Clearly, further studies are needed to evaluate this hypothesis and analyze the adjuvant’s mechanism of action.

Interestingly, pwMS during S1PR modulation rather developed anti-SARS-CoV-2 antibody responses after NVX-CoV2373 immunization, whereas pwMS under ocrelizumab presented with SARS-CoV-2-specific T-cell responses. Due to peripheral lymphopenia as an essential consequence of the mechanism of action of S1PR modulation, the lack of measurable T-cell responses to vaccination in these patients was not entirely surprising. Concerning anti-CD20 treatment, several studies have provided evidence for maintained or even enhanced T-cell responses to SARS-CoV-2 mRNA and viral vector vaccines (22, 23, 27, 50–53). This observation elicited hope that the antivaccine T-cell response may protect affected patients from infection and severe disease courses in the absence of vaccine-specific antibodies. For the reported study, we offered NVX-CoV2373 vaccination only to those patients who had not mounted a sufficient antibody or T-cell response to previous vaccination with mRNA or viral vector vaccines. Encouragingly, the propensity to mount T-cellular vaccination responses in ocrelizumab-treated pwMS was significantly stimulated by NVX-CoV2373, even if previous immunization with other SARS-CoV-2 vaccines had not sufficed. The lack of humoral response even after NVX-CoV2373 vaccination is likely due to the absence of CD20 plasma cell precursors as a consequence of the medication’s mechanism of action.

Our study is limited by the inability to draw conclusions on the clinical efficacy of NVX-CoV2373 vaccination and by the absence of a comparator arm receiving mRNA or vector vaccines as the fourth and fifth booster shots or patients that received only NVC-CoV2373 vaccine as their primary vaccination. So we are not able to completely exclude that immunological effects are induced by the combination of serial vaccinations rather than the vaccination type. As measurements after mRNA/viral vector vaccinations were not part of the standard protocol, the time point between mRNA/viral vector vaccination versus NVX-CoV2373 and blood testing varied, and we cannot exclude that the initial immune response elapsed over time. Moreover, follow-up time points evaluating the dynamics of the T- and B-cellular response later than 8 weeks after NVX-CoV2373 are not available, which is important, especially in immune-modulated/immunosuppressed patients. Moreover, the humoral responses of seven patients could not be included in the analysis due to pre-exposure prophylactic treatment with monoclonal anti-SARS-CoV-2 antibodies, which was initiated during the study period. As discussed above, S1PR-modulated patients did not show a significant increase in T-cell responses after vaccination. Possibly, the QuantiFERON T-cell assay is not sufficiently assessable in these patients due to the medication-induced lymphopenia. However, this problem applies to all assays for the measurement of T-cell reactivity in patients during S1PR modulation.

In conclusion, our data may impact clinical decision-making by offering evidence that immunization with NVX-CoV2373 can lead to an anti-SARS-CoV-2 immune response in pwMS receiving treatment with S1PR modulation or anti-CD20 treatment who failed to mount a response to prior mRNA and/or viral vector vaccination.

## Data availability statement

The raw data supporting the conclusions of this article will be made available by the authors, without undue reservation.

## Ethics statement

The studies involving human participants were reviewed and approved by institutional review board of the University Hospital Dresden. The patients/participants provided their written informed consent to participate in this study.

## Author contributions

CW, TZ, and KA designed the study. MM-E, CW, GK, MD, and KA acquired, analyzed, or interpreted the data. GK provided administrative/technical support. MM-E, CW, and KA drafted the manuscript. GK, RH, MD, and TZ revised the manuscript for important intellectual content. RH performed the statistical analysis. TZ and KA supervised the study. KA has full access to all the data in the study and takes responsibility for the integrity of the data and the accuracy of the data analysis. All authors contributed to the article and approved the submitted version.
